# The IMOVE trial: Behavioral and imaging results of an arts-based intervention in people with AD

**DOI:** 10.1093/geroni/igaf122.3665

**Published:** 2025-12-31

**Authors:** Christina Hugenschmidt, Jason Fanning, Hannah Menaker, Santiago Saldana, Deepthi Thumuluri, Jessie Laurita-Spanglet, Edward Ip, Christina Soriano

**Affiliations:** Wake Forest University School of Medicine, Winston-Salem, North Carolina, United States; Wake Forest University, Winston-Salem, North Carolina, United States; Wake Forest University School of Medicine, Winston-Salem, North Carolina, United States; Wake Forest University School of Medicine, Winston-Salem, North Carolina, United States; Wake Forest University School of Medicine, Winston-Salem, North Carolina, United States; Wake Forest University, Winston-Salem, North Carolina, United States; Wake Forest University School of Medicine, Winston-Salem, North Carolina, United States; Wake Forest University, Winston-Salem, North Carolina, United States

## Abstract

The IMOVE study was a randomized, single-blind trial in dyads (n = 101) of people with MCI or early-stage Alzheimer’s disease (PWAD) and their caregivers. IMOVE used a factorial design with four arms designed to test a Movement factor (MOV) and a Social Engagement factor (SOC): the Movement Group (MG) used a dance intervention (MOV+/SOC+); the Movement Alone (MA) arm used the same dance stimulus, but not in a group (MOV+/SOC-); the Social Group (SG) included arts-based social games (MOV-/SOC+); and a Usual Care (UC) control arm (MOV-/SOC-). The primary outcome was quality of life (QOL). Secondary outcomes included physical function, mood, cognition, and structural and functional brain imaging. Behavioral outcomes were assessed using nonparametric methods. Brain imaging outcomes were tested in SPM. Models tested the effects of factors, interactions between factors, and switch to virtual intervention in response to COVID. No significant effects of MOV or SOC were observed for QOL. For secondary outcomes, randomization to a MOV study arm resulted in significant improvements in postural sway on a foam mat (P < 0.05) and the Fullerton Advanced Balance Scale (P < 0.05). Randomization to a SOC study arm resulted in improved cognitive performance using the RBANS (P < 0.05) and depression scores (P < 0.05). Diffusion imaging results showed associations with MOV and SOC factors and their interactions in the superior longitudinal fasciculus, posterior limb of the internal capsule, and corticospinal tracts. Overall results suggest that 12 weeks of arts-based movement and social engagement may have separate and beneficial effects in PWAD.

